# Isolation and Identification of Ferulic Acid From Aerial Parts of *Kelussia odoratissima* Mozaff.

**Published:** 2012-10-07

**Authors:** Seyed Ebrahim Sajjadi, Yalda Shokoohinia, Narjess-Sadat Moayedi

**Affiliations:** 1 Department of Pharmacognosy, Faculty of Pharmacy, Isfahan University of Medical Sciences, Isfahan, IR Iran; 2 Department of Pharmacognosy and Biotechnology, School of Pharmacy, Kermanshah University of Medical Sciences, Kermanshah, IR Iran; 3 Isfahan Pharmaceutical Sciences Research Center, Isfahan University of Medical Sciences, Isfahan, IR Iran

**Keywords:** *Kelussia odoratissima*, Ferulic Acid, Isolation

## Abstract

**Background:**

*Kelussia odoratissima* Mozaff. is one of the newest genera of Umbelliferae which is represented by only one species. This sweet-smelling, self-growing monotypic medicinal plant is endemic to a restricted area in west of Iran and is locally called Karafse-koohi. The aerial parts of the plant are commonly used as a popular garnish and a sedative medicinal plant. There are several reports concerning antioxidant, anti-inflammatory, anxiolytic and hypolipidemic activities of aerial parts of *K. odoratissima*.

**Objectives:**

The current research aimed to evaluate some phenolic contents of the plant for the first time .It is confirmed that secondary metabolites and especially phenolic compounds have important role in the biological activities of the plant. Available information indicates that phenolic contents of *K. odoratissima* have not been the subject of any investigation

**Material and Methods:**

Aerial parts of *K. odoratissima* were extracted with acetone by maceration method. Normal and reversed phase vacuum liquid chromatography used to fractionate the extract. ^1^H-NMR, ^13^CNMR, EI-Mass and IR spectra were used to elucidate isolated compound.

**Results:**

The phenolic acid isolated compound was identified as 4-hydroxy-3-methoxycinnamic acid (ferulic acid).

**Conclusions:**

Compared with previous reported antioxidant and anti-inflammatory properties of ferulic acid, a chemical-biological relation can be postulated.

## 1. Background

Umbelliferae includes more than 275 genera and 2850 species all around the world (1). *Kelussia* is one of the newest genera of this family and is represented by only one species, *Kelussia odoratissima* Mozaff., which is found only in Iran (2). This sweet-smelling, self-growing monotypic medicinal plant is endemic to a restricted area in western Iran and is locally called “Karafse-koohi”. The aerial parts of the plant are commonly used as a popular garnish and a sedative medicinal plant.

The antioxidant activity of the methanolic extract of the plant was evaluated by β-carotene bleaching assay, reducing power, thiocyanate, accelerated oxidation of sunflower oil, and DPPH radical-scavenging which was effective in some assays (3, 4). In spite of its in vitro and *in vivo* efficiencies in lipid profile (5) the results could not be confirmed in a clinical trial, except for an increase in HDL (6). Furthermore, feeding rabbits with the aerial parts of the plant, suggested the existence of beneficial effects to prevent development of fatty streak (7). Besides, gastric acid secretion has been reduced meaningfully in rats fed with *K. odoratissima*, but pepsin secretion was unaffected (8). Essential oil of the plant showed a pronounced antibacterial effect (9) and folkloric proposed use of the plant as anti-inflammatory agent was confirmed via a carrageenan test (10).

## 2. Objectives

The current research aimed to isolate and elucidate ferulic acid from *K. odoratissima* of the plant for the first time in continuation of the phytochemical and pharmacological investigations on this new species of Umbelliferae (11).

## 3. Materials and Methods

### 3.1. Plant Material

Aerial parts of *K. odoratissima* were collected from Zardkooh Mountain, Charmahal Bakhtiari province (south western Iran) in March 2011. The plant identity was confirmed by the Botany Department of Isfahan University. A voucher specimen of plant was deposited at the herbarium of the school of Pharmacy and Pharmaceutical Sciences, Isfahan University of Medical Sciences, Isfahan, Iran (No. 2022).

### 3.2. Ferulic acid Isolation

The air dried aerial parts of *K. odoratissima* was ground to the coarse powder (340g) and extracted with acetone (3.4 L) by maceration method (one day soaking; three times extraction). The extract was concentrated by rotary evaporator to obtain a green viscose mass (EXT1 = 25.4 g). The crud extract was filtered on a bed of silicagel using heptane- EtOAC resulting in 18.2g filtrate (EXT2). The EXT2 was fractioned by Vacuum Liquid Chromatography (VLC) (Silicagel RP_18_) using a gradient mobile phase of MeOH: H_2_O from 60 to 100% to afford seven fractions (A2-G2).

The fractions of A2, B2 and C2 were pooled according to TLC profile by cerium sulfate molybdate as chromatographic reagent and were dried by rotary evaporator. Later, the dried samples were separated by a normal phase VLC (Silicagel; 0.015-0.04 mm) using heptane- EtOAC (7:32:8) to obtain eight fractions (A3-H3), among them, fraction F3 rendered mass of light yellow crystals which was subject to recrystallization process until resulted pure crystalline (304mg). Other fractions were discarded.

### 3.3. Analytical Instruments

The IR spectrum was recorded on a Rayleigh WQF-510 FTIR instrument. ^1^ H-NMR was recorded on Bruker (500 MHz) instrument. EI-MS spectrum was recorded on Agilent 5975C mass spectrometer.

### 3.4. Materials

CDCl3 (Merck) and TMS (Merck) were used as solvent and internal standard respectively in NMR analyses. Silicagel 60 GF_254_ pre-coated plates (Merck), cerium sulfate molybdate as spraying reagent and other solvents and solid materials were also purchased from Merck (Germany).

## 4. Results

**Figure 1:**
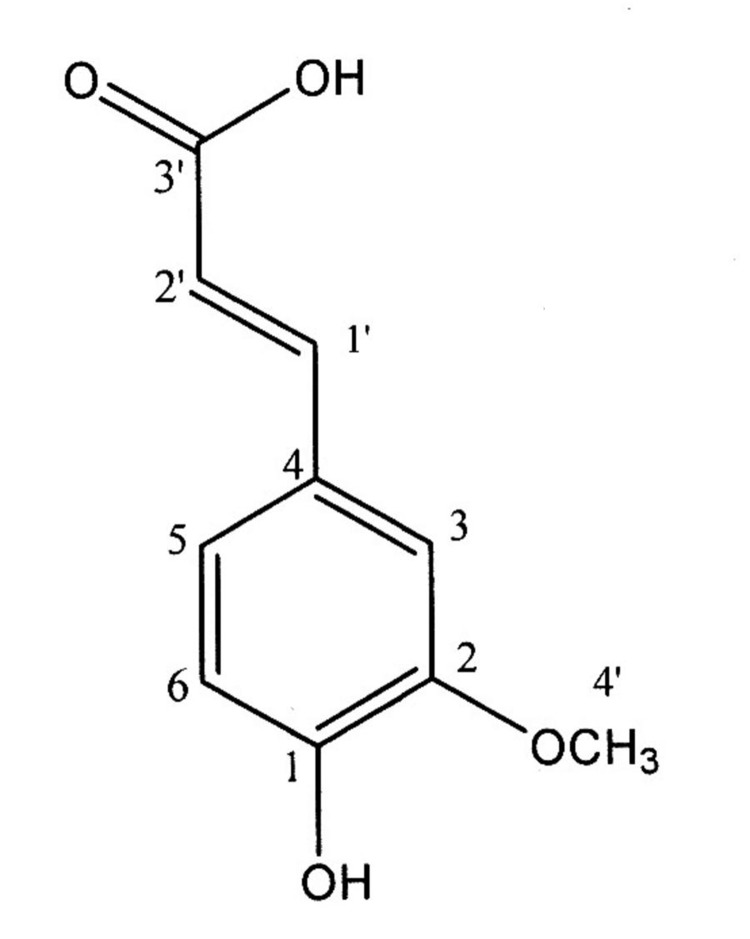
Structure of Ferulic acid

Light yellow crystals, obtained from F3 fraction, were subject to recrystallization process until resulted 304mg pure crystalline material. The structure of isolated compound with melting point of 168° C is presented in Figure 1. The isolated compound was elucidated by, ^1^HNMR, ^13^CNMR, IR and MS as well as comparison of the data with those reported in the literature (12). The analytical data were as follows:

**^1^HNMR : **^1^HNMR (500 MHz, CDCl3, J in Hz) δ: 3.98 (3H, s, H-4’), 6.34 (1H, d, J=15 Hz, H-2’), 6.97 (1H, d, J=9 Hz, H-6), 7.14 (1H, dd, J=8 and 2 Hz, H-5), 7.09 (1H, d, J=2 Hz, H-3), 7.75 (1H, d, J=15 Hz, H-1’).

^1^HNMR spectrum displayed the characteristic signal for a methoxy group at δ 3.98 (s). The compound spectrum also showed three aromatic proton at δ 6.97 (d; J=9 Hz), 7.14 (dd; J=8 and 2 Hz) and 7.09 (d; J=2 Hz), characteristics for the H-6, H-5 and H-3 of aromatic part of isolated compound. The presence of further two proton doublets with J= 15 Hz at δ 6.34 and 7.75 indicated the presence of H-2’ and H-1’ in the side chain of compound respectively.

**^13^CNMR: **^13^CNMR (500 MHz, CDCl3) δ: 55.98 (C-4’), 109.48 (C-5), 114.39 (C-2), 114.78 (C-2’), 123.57 (C-3), 126.68 (C-4), 146.81 (C-1’), 147.05 (C-6), 148.37 (C-1), 171.36 (C-3’).

The ^13^CNMR spectrum showed the presence of 10 signals (6 aromatic carbon and 4 aliphatic chain) in agreement with the proposed structure of ferulic acid (4-hydroxy-3-methoxycinnamic acid) (Figure 1).

**Mass:** EI-MS m/z (rel. int.): 194 [M]+ (100), 179 (21), 161 (7), 133 (32), 105 (14), 89 (15), 77(27), 51(15); FT-IR (KBr): νmax = 3450, 1690, 1605, 1510, 1275, 940 cm-^1^. The EI-MS showed a molecular ion peak at m/z 194 (M+, base peak) in agreement with the proposed structure of the known phenolic compound, ferulic acid, with C10H10O4 molecular formula.

**Figure 2:**
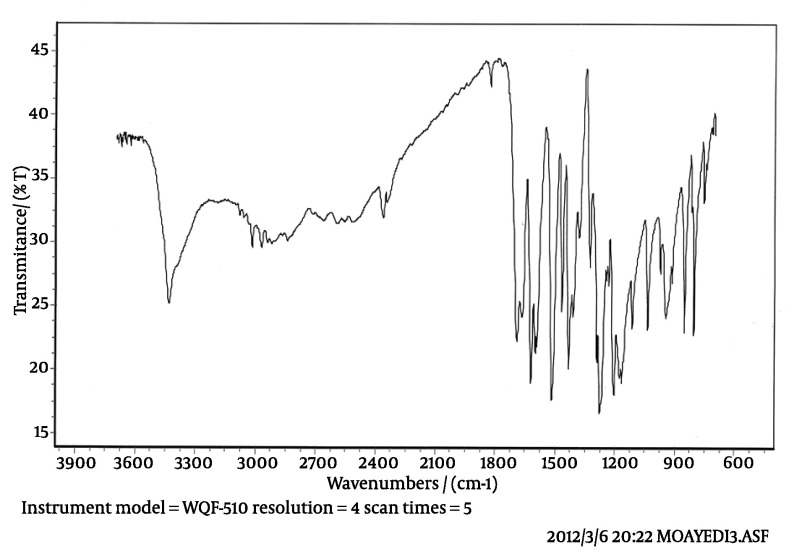
IR spectrum of isolated compound

**Figure 3:**
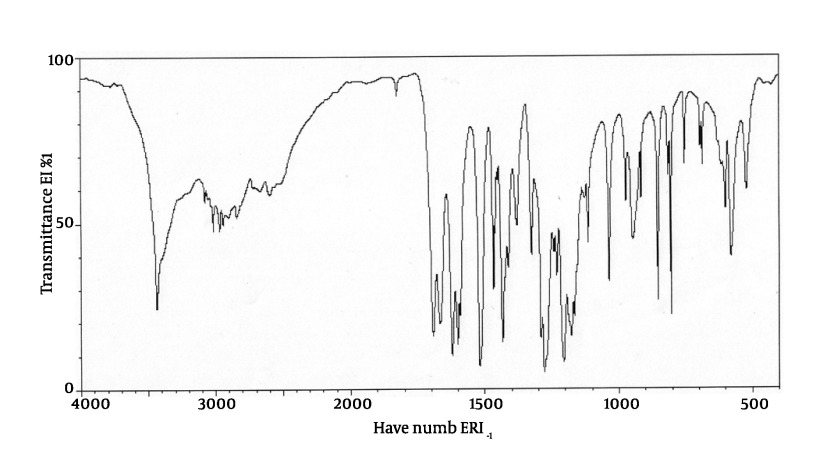
IR spectrum of Ferulic acid

**IR:** The IR spectrum with the peaks at 3450 cm-^1^ (carboxylic acid O-H stretching), 1690 cm-^1^ (carboxylic acid C=O stretching), 1275 cm-^1^ (carboxylic acid C-O stretching) and 1510; 1605 cm-^1^ (aromatic C=C) confirms the skeleton of ferulic acid. The IR spectrum of isolated compound and standard spectrum of ferulic acid are presented in Figure 2 and Figure 3 respectively. As it can be concluded, the IR spectrum of isolated compound is completely in agreement with the proposed structure of ferulic acid.

## 5. Discussion

Coumarins, phenolics, flavonoids, terpinoids, phthalides and essential oils are important chemical constituents of Umbelliferae (1, 13, 14). Ferulic acid (4-hydroxy-3-methoxycinnamic acid), is a phenolic acid compound which arises from the metabolism of phenylalanine by shikimic acid pathway in plants. Due to its phenolic nucleus and extended conjugated side chain, ferulic acid is an important natural potent antioxidant (15), so it has been approved in some countries as food additive to prevent lipid peroxidation (16). Furthermore, there are many reports on antimicrobial activity of ferulic acid, thus, it can be used as a natural preservative (17).

Antioxidant activity of ferulic acid resulted in pharmacological activities, including anti-cancer, anti-inflammatory and protective effect against coronary heart disease (17). There is another report on inhibitory effect of this low molecular weight natural phenolic compound on tumor promotion of mouse skin (18). Since *K. odoratissima* is an edible plant in Iran, isolation and identification of any pharmacological active component could be very important. Volatile constituents of *K. odoratissima* were reported by the authors in advance. The main constituents of the volatile oil are phthalides with z-ligustilide as the major constituent (11). However, to the best of authors knowledge, this paper was the first report on non-volatile components of *K. odoratissima*. As mentioned in the introduction, it has been confirmed that the aerial parts of this edible plant possess antioxidant and anti-inflammatory activities (3, 10). Because of antioxidant and anti-inflammatory properties of ferulic acid, a chemical-biological relation can be postulated.
